# Optimizing Health and Sustainability: Innovations in Harnessing Phytochemicals for Functional Foods

**DOI:** 10.1002/fsn3.71002

**Published:** 2025-09-22

**Authors:** Faiyaz Ahmed, Sammra Maqsood, Md Faruque Ahmad, Ahmadullah Zahir

**Affiliations:** ^1^ Department of Basic Health Sciences College of Applied Medical Sciences, Qassim University Buraydah Saudi Arabia; ^2^ National Institute of Food Science and Technology University of Agriculture Faisalabad Faisalabad Pakistan; ^3^ Department of Clinical Nutrition, College of Nursing and Health Sciences Jazan University Jazan Saudi Arabia; ^4^ Faculty of Veterinary Sciences, Department of Food Science and Technology Afghanistan National Agricultural Sciences and Technology University Kandahar Afghanistan

**Keywords:** bioactive compounds, bioavailability, extraction method, health, phytochemicals

## Abstract

Phytochemicals, bioactive compounds of plant sources, have drawn considerable interest in research and development in the field of functional foods owing to their potential health impacts and importance in sustainable food. This review deliberates the modern advances in employing phytochemicals to improve food production sustainability and enhance health. Improvements in phytochemical extraction and processing, their addition to functional food products and their usage in addressing global public health glitches such as cardiovascular disease, metabolic disease, and cognitive impairment are important areas. The paper also highlights the role of circular food economies, sustainable food systems, and food waste valorization in exploiting the usage of phytochemicals. Problems of bioavailability, regulatory acceptance, and consumer acceptance persist despite their massive potential. In understanding the full potential of phytochemicals in functional foods, upcoming efforts will be intended at interdisciplinary investigation into consortia, personalized nutrition, and artificial intelligence‐driven food invention. This assessment highlights the need for a sustainable food system and explores the findings to improve the longevity of both humans and the environment.

## Graphical abstract

Advances in phytochemical extraction and integration into functional foods are reviewed. Phytochemicals help address cardiovascular, metabolic, and cognitive health challenges. Circular food systems and waste valorization enhance sustainable use of phytochemicals. Future focus includes bioavailability, regulation, AI‐driven innovation, and personalization.

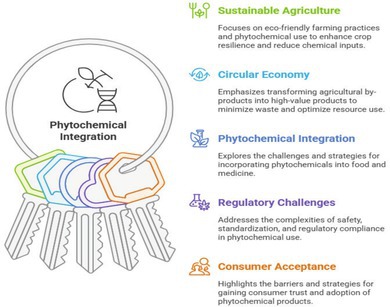

## 1 Introduction

Natural occurring bioactive composites known as phytochemicals deliver plants with color, flavor, and resistance to environmental stresses. Well‐studied health beneficial physiognomies of these composites such as alkaloids, polyphenols, flavonoids, and carotenoids have been extensively investigated. Due to antioxidative, anti‐inflammatory, and disease‐averting activities, phytochemicals are critical constituents of functional foods and play a significant role in human nutrition (Kussmann et al. 2023). Phytochemicals are being added to purposeful meals to attain the utmost level of nutrition and sustainability due to developments in food science resulting from improved knowledge of their health assistances (Granato et al. 2020). Superfoods are nutrient‐dense foods—often plant‐based but also including some fish and dairy—considered especially beneficial for health and well‐being due to their high concentration of vitamins, minerals, antioxidants, and other bioactive compounds (Fernández‐Ríos et al. 2022). Lesser recognized phytochemical‐rich foods like Sacha Inchi and some forms of algae are said to improve health and well‐being (Yadav and Yadav 2024; Wells et al. 2017). Such foods comprise substantial levels of proteins, biologically active components, and vital fatty acids that improve immunological and metabolic well‐being (Valdez‐Arana et al. 2025). In addition, a circular economy strategy for sustainability has been advanced by integrating food industry by‐products in the formulation of functional foods and reducing waste while augmenting nutritional content (Ospina‐Maldonado et al. 2024). To contest oxidative stress and long‐term illnesses, carotenoids—a group of phytochemicals extensively studied for their antioxidant properties—have been added to functional foods and nutraceuticals (Meléndez‐Martínez et al. 2021). They are appreciated ingredients in novel food preparations due to their capability to counteract inflammation and advance the health of the skin and eyes. Just like this, polyphenols—which are present in important amounts in honey, strawberries, and olives—own powerful chemopreventive and anti‐inflammatory characteristics that subsidize augmenting health and disease anticipation (Battino et al. 2021; Gouvinhas et al. 2017). The gut flora intercedes the health influence of dietary phytochemicals to a great extent. Studies demonstrate how particular biological compounds impact the conformation of microorganisms, improving gut health and immune response (Jacquier et al. 2024; Yin et al. 2019). The functional cooperation among microbiota and dietary phytochemicals is also exemplified by postbiotics and parabiotics derived from probiotic fermentation, which augments and improves metabolic and digestive consequences (Nataraj et al. 2020). How consumers distinguish functional foods also shows a great role in their extent of adoption into diets. Investigation finds that insights towards functional foods are diverse across sections and what customers judge and choose is a function of dimensions such as flavor, convenience, and health assertions (Nystrand and Olsen 2020; Küster‐Boluda and Vidal‐Capilla 2017). To certify customer confidence, regulatory measures are also vital in certifying that functional food products are nontoxic and operative, for which stringent labeling necessities are required (Díaz et al. 2020).

**FIGURE 1:**
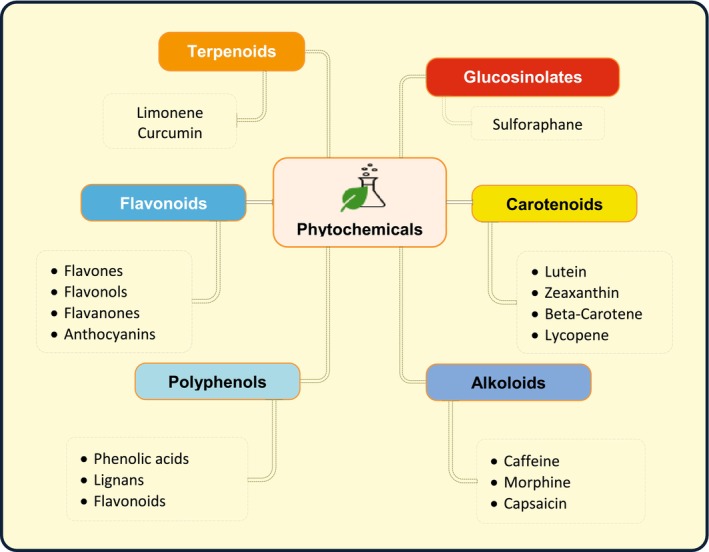
Diagram showing major classes of phytochemicals organized around a central node, with representative compounds listed for each class: Terpenoids (limonene, curcumin), glucosinolates (sulforaphane), carotenoids (lutein, zeaxanthin, beta‐carotene, lycopene), flavonoids (flavones, flavonols, flavanones, anthocyanins), polyphenols (phenolic acids, lignans, flavonoids), and alkaloids (caffeine, morphine, capsaicin).

Integrating phytochemicals into functional foods presents a supportable solution for health improvement. Developments in food science continually determine new therapeutic compounds that augment human well‐being and diminish their adverse influences on the environment. For the elevation of informed customer choice, future investigation must focus on intensifying the number of functional foods accessible, exploiting their bioavailability, and strengthening regulatory systems. An enhanced and maintainable food system is attained by comprising phytochemicals in daily meals, which can develop preventative care (Adefegha 2018; Ranjan et al. 2019). Phytonutrients play an acute role in several physiological procedures comprising anti‐inflammatory action, antioxidant capability, and modification of cellular paths linked with chronic disease (Battino et al. 2021). Phytochemical antioxidant capability is one of the most well studied of their effects. Age and numerous chronic diseases such as cancer and cardiovascular diseases are deteriorated by oxidative stress, which is produced by an imbalance among the antioxidants and free radicals in the body (Kim and Lee 2021). Secondary plant metabolites such as flavonoids, carotenoids, and polyphenols decrease oxidative damage and augment cellular well‐being by neutralizing free radicals (Monjotin et al. 2022). Neurodegenerative diseases, cardiovascular disease, and arthritis all segment a link with chronic inflammation. Inhibition of inflammatory mediators and paths is consummate by numerous phytochemicals that exhibit anti‐inflammatory activity. For instance, polyphenols present in fruits and vegetables decrease the incidence of chronic diseases by regulating inflammatory reactions (Gouvinhas et al. 2017). Additionally, the materials augment immunity, making the body more resilient to diseases and infections (Yin et al. 2019). The anti‐cancer potential of phytochemicals has been comprehensively researched. According to Singh et al. (2017), functional biomaterials like isothiocyanates, flavonoids, and saponins have been promising in hindering tumor development, persuading apoptosis, and restricting cancer cell reproduction. Based on investigation, consumption of foods with high phytochemical contents may decrease the incidence of assured forms of cancer, comprising colorectal, prostate, and breast cancer (George et al. 2021). Numerous plant‐derived compounds lower blood pressure, cholesterol, and hinder atherosclerosis, all causative to better cardiovascular health. Flavonoids and polyphenols found in berries, tea, and olive oil are accountable for endothelial function improvement and abridged heart disease risk (Díaz et al. 2020). The cardioprotective properties of microalgae‐based omega‐3 fatty acids have also been documented (Matos et al. 2017). The gut microbiota conformation is resolved by dietary phytochemicals, which decrease pathogenic bacteria and arouse the development of beneficial bacteria. Yin et al. (2019) explain that this interaction improves digestion, nutrient uptake, and general gut health. Probiotics are crucial for sustaining a normal gut microbiota, and their expansion is boosted by fiber‐based phytochemicals present in fruits, vegetables, and whole grains (Adefegha 2018). Neuroprotection and improvement of cognitive function have been associated with phytochemicals. By averting neurons from oxidative stress and inflammation, compounds such as flavonoids and polyphenols recover learning and memory (Battino et al. 2021). Evidence suggests that diets rich in phytochemicals lower the incidence of neurodegenerative diseases like Parkinson's and Alzheimer's (Ahmed et al. 2017). Functional food demand has risen as a concern of improved awareness of the benefits of phytochemicals. As per Küster‐Boluda and Vidal‐Capilla (2017), customers prefer meals that are supplemented with bioactive substances with health benefits in addition to mere nourishment. Functional food cataloging and entitlements have been created as strategies due to regulatory bodies across the globe understanding the prominence of phytochemicals (Díaz et al. 2020). Due to their anti‐inflammatory, anti‐cancer, cardiovascular, and antioxidant activities, phytochemicals are vigorous to human well‐being and classified in various categories (Figure 1). Their addition to daily diets, mainly via functional foods, augments overall wellness and averts chronic diseases. To fully recognize phytochemical mechanisms and potential applications in current nutrition and medicine, there is a need for supplementary investigation.

### 1.1 Aim of the Review

The general purpose of this review is to deliberate the latest advances in the usage of phytochemicals towards sustainable food systems and health expansion. This review delivers an insightful consideration of how phytochemicals assist with disease anticipation and general well‐being by bearing in mind cutting‐edge extraction processes, functional food formats, and their particular health benefits. In addition, it highpoints how phytochemicals contribute to maintainable agriculture and circular food economies, which decrease waste and augment sustainability in the environment. Through this review, a future line of investigation and novelty in functional foods based on phytochemicals is existing by addressing areas of bioavailability, regulatory protection, and customer acceptance.

## 2 Understanding Phytochemicals

Plants have bioactive composites known as phytochemicals which play a critical role in plant metabolism and exert important beneficial impacts on human health. Although not vital nutrients, these composites avert disease and improve overall health (Bocso and Butnariu 2022). Polyphenols, flavonoids, carotenoids, alkaloids, glucosinolates, and terpenoids are some of the phytochemicals that are categorized based on their biochemical structure and function (Lobo et al. 2018). They form a precarious part of human diets because they can be present in fruits, vegetables, herbs, and whole grains. They are also anti‐inflammatory, anti‐cancer, and antioxidant in natural surroundings (Ahmed et al. 2017).

### 2.1 Classification of Phytochemicals

#### 2.1.1 Polyphenols

One of the major groups of phytochemicals, polyphenols is also notorious for their potent antioxidant and anti‐inflammatory properties. According to Francisco et al. (2017), they are further partitioned into phenolic acids, lignans, and flavonoids. Flavonoids such as quercetin and catechins, present in tea, apples, onions, and berries, are associated with reduced risks of cardiovascular disease and neurodegenerative diseases at reduced risks (Thakur and Sharma 2018). Vegetables, whole grains, and coffee are outstanding sources of caffeic acid and ferulic acid, phenolic acids that own resilient antioxidant activity (Lima et al. 2017). Lignans present in seeds, chiefly in flaxseeds, assist in hormonal steadiness and can confer defense against malignancies that have a hormonal constituent, as stipulated by Nwozo et al. (2023).

#### 2.1.2 Flavonoids

Plant coloration and human health assistance are due to flavonoids, which are an extensive group of polyphenolic compounds (Ahmed et al. 2017). They all have different physiological actions and consist of flavones, flavonols, flavanones, and anthocyanins (Ahangarpour et al. 2019). For example, anthocyanins present in red cabbage and blueberries have been related to enhanced cardiovascular and cognitive well‐being (Sharma et al. 2019). Leafy greens and berries comprise flavonols such as myricetin and kaempferol, which have been related to augmented immune function and reduced inflammation (Napoli et al. 2018).

#### 2.1.3 Carotenoids

Carotenoids are lipid‐soluble pigments liable for the red, orange, and yellow colors of most fruits and vegetables (Mollakhalili Meybodi et al. 2017). These antioxidant and vision‐defensive composites contain of lutein, zeaxanthin, beta‐carotene, and lycopene (Engwa 2018). Beta‐carotene, present in carrots and sweet potatoes, augments skin and immunological health and aids as a precursor to vitamin A (Wang et al. 2018). Tomatoes and watermelon are good sources of lycopene, which is associated with an abridged risk of prostate cancer as well as cardiovascular disease (Singh et al. 2018).

##### 2.1.3.1 Alkaloids

Alkaloids, which are composites that comprise nitrogen and have pharmacological activities like analgesia, inflammation reticence, and antibacterial properties, render to Ahmed et al. (2017). They are found in plants such as tea, coffee, and some medicinal herbs. Tea and coffee comprise caffeine, a conversant alkaloid that improves metabolism and intellectual function (Ahangarpour et al. 2019). The opium poppy plant yields morphine, which is also usually used for pain relief (Uddin et al. 2020). Other alkaloids, like capsaicin in chili peppers, own thermogenic properties to benefit from weight loss, says Zahedipour et al. (2022).

#### 2.1.4 Glucosinolates

As per Francisco et al. (2017), cruciferous vegetables such as broccoli, cabbage, and Brussels sprouts are chiefly liable for comprising glucosinolates, which are sulfur‐rich substances. These composites are hydrolyzed to yield bioactive isothiocyanates, which have been established to exhibit anti‐cancer properties by constraining tumor growth and commencing apoptosis (Singh et al. 2018). One of the extensively investigated isothiocyanates, sulforaphane, is highly well‐known for its role in detoxification and defense against diseases related to oxidative stress (Chen et al. 2018).

#### 2.1.5 Terpenoids

Terpenoids, also named isoprenoids, are an extensive group of chemical molecules that confer flavor and smell to various plants (Francisco et al. 2017). They encompass the antibacterial, anti‐inflammatory, and anticancer actions of monoterpenes, sesquiterpenes, and diterpenes (Khan et al. 2020). Citrus fruits are rich in limonene, which has been established to have potential chemopreventive activities (Hannan et al. 2020). Turmeric's curcumin diterpenoid has been widely investigated to have neuroprotective and anti‐inflammatory properties (Ong et al. 2017) (Table 1).

**TABLE 1:** Phytochemicals in functional foods: Innovations in health optimization and sustainable food systems.

Phytochemical class	Example compounds	Sources	Antioxidant properties	Modulation of signaling pathways	Epigenetic effects	References
Polyphenols	Resveratrol, Quercetin, Catechins	Grapes, Berries, Green Tea	Scavenges free radicals, reduces oxidative stress	Anti‐inflammatory via NF‐κB inhibition, neuroprotective	DNA methylation alterations	Furrer et al. (2018); Lobo et al. (2018)
Flavonoids	Kaempferol, Apigenin, Luteolin	Citrus fruits, Onions, Parsley	Inhibits lipid peroxidation	Modulates MAPK and PI3K/Akt pathways	Affects histone acetylation	Bocso and Butnariu (2022); Ahmed et al. (2017)
Carotenoids	Beta‐carotene, Lycopene, Lutein	Carrots, Tomatoes, Spinach	Quenches singlet oxygen	Anti‐inflammatory via Nrf2 activation	Epigenetic modulation of inflammatory genes	Abdelkhalek et al. (2024); Lima et al. (2017)
Alkaloids	Caffeine, Theobromine, Berberine	Coffee, Cocoa, Goldenseal	Reduces ROS‐induced damage	Affects neurotransmitter signaling	Modulates DNA methylation	Alamgir and Alamgir (2018); Sharma et al. (2019)
Glucosinolates	Sulforaphane, Indole‐3‐carbinol	Broccoli, Brussels Sprouts, Kale	Enhances cellular antioxidant defense	Modulates Nrf2 and p53 pathways	Promotes histone deacetylase inhibition	Francisco et al. (2017); Nawaz and Shad (2018)
Phenolic Acids	Caffeic Acid, Ferulic Acid	Whole Grains, Coffee, Apples	Neutralizes free radicals	Regulates pro‐inflammatory cytokines	Influences histone methylation	Amorati and Valgimigli (2018); Engwa (2018)
Isoflavones	Genistein, Daidzein	Soybeans, Legumes	Reduces oxidative DNA damage	Estrogen receptor modulation	Alters miRNA expression	Nawaz et al. (2020); Uddin et al. (2020)
Tannins	Ellagitannins, Proanthocyanidins	Pomegranates, Tea, Grapes	Chelates metal ions, reduces ROS	Anti‐inflammatory via COX‐2 inhibition	Modifies chromatin remodeling	Parcheta et al. (2021); Akhtar and Mirza (2018)
Terpenoids	Limonene, Curcumin, Carvacrol	Citrus Peels, Turmeric, Oregano	Scavenges peroxyl radicals	Regulates NF‐κB and JAK–STAT pathways	Modulates histone phosphorylation	Napoli et al. (2018); Ong et al. (2017)
Saponins	Diosgenin, Ginsenosides	Legumes, Ginseng, Quinoa	Prevents oxidative LDL modification	Inhibits pro‐inflammatory mediators	Affects histone deacetylation	Irakli et al. (2019); Singh et al. (2018)
Anthocyanins	Cyanidin, Delphinidin	Berries, Red Cabbage	Enhances glutathione levels	Downregulates inflammatory genes	Alters DNA methylation patterns	Wang et al. (2018); Sharma et al. (2023)
Lignans	Secoisolariciresinol, Matairesinol	Flaxseeds, Sesame Seeds	Protects against oxidative stress	Phytoestrogenic effects	Histone modification regulation	Chen et al. (2018); Zam and Khadour (2017)
Sterols	Beta‐sitosterol, Campesterol	Nuts, Seeds, Whole Grains	Reduces lipid oxidation	Modulates cholesterol metabolism	Regulates DNA methylation	Gupta et al. (2020); Saleh et al. (2021)
Coumarins	Umbelliferone, Esculetin	Citrus Fruits, Celery, Parsley	Reduces oxidative DNA damage	Suppresses pro‐inflammatory cytokines	Induces histone acetylation	el Omari et al. (2021); Hsieh et al. (2025)
Furanocoumarins	Bergapten, Psoralen	Citrus Peels, Figs	Photoprotective antioxidant effects	Affects cytochrome P450 enzymes	Alters epigenetic signaling	Khan et al. (2024); Arora et al. (2019)
Organosulfur compounds	Allicin, Diallyl Sulfide	Garlic, Onions, Leeks	Enhances glutathione activity	Modulates NF‐κB and MAPK pathways	Affects DNA demethylation	Penta et al. (2018); Dincer and Yuksel (2021)
Phytosterols	Stigmasterol, Campesterol	Nuts, Whole Grains	Prevents lipid peroxidation	Modulates inflammatory pathways	Influences histone methylation	Laboukhi‐Khorsi et al. (2017); Pogorzelska‐Nowicka et al. (2024)

To best comprehend the health benefits of phytochemicals it is essential to understand their taxonomy. Due to their anti‐inflammatory, anti‐cancer and antioxidant properties, these bioactive composites have the capability to ward off disease (Ahmed et al. 2017). The significance of a diet rich in plant foods is brought into attention by the exceptional therapeutic prospective that polyphenols, flavonoids, carotenoids, alkaloids, glucosinolates and terpenoids all possess (Sharma et al. 2019). Their purposes and applications in nutraceuticals and functional meals will be discovered further in subsequent studies (Engwa 2018).

## 3 Mechanisms of Action of Phytochemicals

### 3.1 Antioxidant Properties

Outstanding antioxidant properties are a common piece of phytochemicals, and they play a serious role in defending cells from damage caused by oxidative stress. Cellular dysfunction and the expansion of disease are the concerns of oxidative stress, which occurs when the body produces more reactive oxygen species (ROS) than it can neutralize (Engwa 2018). Phytochemicals' capability to donate electrons neutralizes free radicals and hinders lipid peroxidation, which is the derivation of their antioxidant activity (Amorati and Valgimigli 2018). Carotenoids, flavonoids, and polyphenols possess strong free radical scavenging activity that expressively decreases oxidative stress and the resultant in diseases (Nawaz and Shad 2018). The molecular structure of phytochemicals, predominantly the existence of hydroxyl groups to permit electron donation, has a noteworthy influence on their antioxidant properties. For example, since they can chelate metal ions that catalyze oxidative reactions and stabilize ROS, flavonoids such as quercetin and catechins exhibit high antioxidant activity (Parcheta et al. 2021). Also, phytochemicals improve the antioxidant defense of endogenous antioxidant enzymes such as glutathione peroxidase (GPx) and superoxide dismutase (SOD) by augmenting their activity (Mumivand et al. 2017). The role of phytochemical antioxidants in food preservation and in medicine is an energetic characteristic. Phytochemicals are crucial in antioxidants as they can proficiently avert lipid oxidation in food items and augment their shelf life and nutritional properties (Akhtar and Mirza 2018). Dietary intake of phytochemical‐rich foods like fruits, vegetables, and whole grains has also been associated with a reduced risk for developing chronic conditions like cardiovascular diseases and neurological diseases (Sytar et al. 2018). The capability of phytochemicals to act as natural antioxidant mediators in pharmaceutical products has also been highlighted by current advances. Studies have shown that integrating antioxidants from plants into drug delivery systems improves stability and therapeutic activity (Napoli et al. 2018). Thus, for phytochemicals to be exploited proficiently in health and disease prevention, it is significant to comprehend the molecular basis of their antioxidant activity.

### 3.2 Modulation of Signaling Pathways

Through the modulation of numerous cellular signaling pathways, mainly those linked with inflammation, neuroprotection, and immunological response, phytochemicals utilize their biological actions. These bioactive composites exert anti‐inflammatory and neuroprotective activities through moderating key molecular targets such as nuclear factor kappa B (NF‐κB), mitogen‐activated protein kinases (MAPKs), and nuclear factor erythroid 2‐related factor 2 (Nrf2) (Singh et al. 2018). While inflammation is a vigorous immune response, chronic inflammation has been associated with numerous clinical diseases comprising cancer, cardiovascular disease, and neurodegeneration. It has been revealed that certain phytochemicals, such as curcumin, resveratrol, and epigallocatechin gallate (EGCG), constrain NF‐κB, a key transcription factor intricate in the manifestation of inflammatory genes from being activated (Wang et al. 2018). These composites decrease inflammation‐associated diseases by constraining the manufacture of pro‐inflammatory cytokines by overwhelming NF‐κB signaling (Uddin et al. 2020). Phytochemicals' neuroprotective properties are mainly mediated by their capability to activate the Nrf2‐ARE pathway, which controls antioxidant defenses in neural cells and improves brain‐derived neurotrophic factor (BDNF) signaling (Hannan et al. 2020). Numerous studies have established that phytochemicals are capable of dropping neuroinflammatory responses through the inhibition of extreme fabrication of interleukin‐6 (IL‐6) and tumor necrosis factor‐alpha (TNF‐α), which have been linked with neurodegenerative illnesses such as Parkinson's and Alzheimer's disease (Subedi et al. 2020). Phytochemicals also affect existence and apoptotic pathways, thus subsidizing their aptitude to be anticancerous. Various bioactive composites act against the JAK/STAT and PI3K/Akt signaling pathways, which are acute cell proliferation and survival modulators (Hung et al. 2020). Dietary polyphenols have been shown to induce apoptosis in cancer cells by overexpressing pro‐apoptotic proteins and constraining anti‐apoptotic proteins (Zhang et al. 2019).

### 3.3 Epigenetic Effects, Including DNA Methylation and Histone Modification

Environmental elements comprising diet and phytochemicals impact on epigenetic modifications that are vigorous for gene regulation. Pop et al. (2019) state that epigenetics refers to heritable gene expression alters that do not include DNA sequence alterations, mainly resulting from mechanisms such as DNA methylation, histone modification, and non‐coding RNA regulation. As stated by Gupta et al. (2020), phytochemicals have arisen as active epigenetic modifiers that converse abnormal epigenetic changes linked with diseases such as cancer and dementia. One important epigenetic procedure that regulates gene expression is DNA methylation, where a methyl group is added to cytosine remains within CpG islands. It has been shown that certain phytochemicals such as genistein, sulforaphane, and epigallocatechin‐3‐gallate (EGCG) overwhelm DNA methyltransferases (DNMTs), which avert tumor suppressor genes in cancer cells from becoming hypermethylated (Zam and Khadour 2017). For instance, sulforaphane from cruciferous vegetables is a potent inhibitor of both DNMT1 and DNMT3a enzymatic activity, leading to the hypomethylation and reactivation of the tumor suppressor gene p16INK4a in colon cancer cells (Hsu et al. 2011). This effect inspires the death of cancer cells through the refurbishment of gene expression related to cell cycle regulation and apoptosis (el Omari et al. 2021). Acetylation and methylation are just two of the histone modifications that play critical roles in gene transcription and chromatin remodeling. Histone acetyltransferases (HATs) and histone deacetylases (HDACs) are moderated by phytochemicals, which impact gene expression and chromatin approachability (Khan et al. 2024). Resveratrol and curcumin, for instance, have been stated by Arora et al. (2019) to upsurge histone acetylation, which consequently triggers tumor suppressor genes and represses oncogenic paths. Curcumin has been shown to directly inhibit the activity of the histone acetyl transferase p300/CBP, a specific member of the HAT family, thereby modulating the acetylation of histones H3 and H4 at promoters of pro‐inflammatory genes. Predominantly, non‐coding RNAs, the long non‐coding RNAs (lncRNAs), and the microRNAs (miRNAs) are now important modulators of gene expression affected by dietary phytochemicals. From studies, it was experiential that curcumin, along with other polyphenols, impacts the expression profile of miRNAs, which affect apoptosis, inflammation, as well as neuroprotection (Zhao et al. 2023). It has the insinuation that numerous diseases will be evaded as well as being treated through exhausting the phytochemicals as part of the epigenetic therapy. All in all, the view of phytochemicals in disease anticipation and therapy is underscored by their capability to impact epigenetic paths. To best understand their therapeutic potential, future investigation should focus on recognizing the specific molecular connections of phytochemicals with epigenetic controllers (Hsieh et al. 2025).

## 4 Advances in Phytochemical Extraction and Processing

**FIGURE 2:**
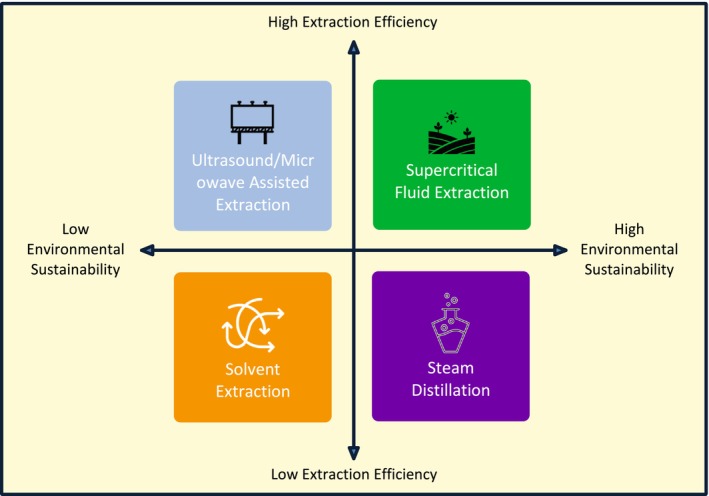
The illustration highlights a few specific methods of extraction that are applied for the evaluation of distinctive phytochemicals. These approaches comprise solvent extraction, steam distillation, supercritical fluid extraction, and ultrasonic extraction. When it comes to effectively accumulating bioactive compounds, each technique offers specific advantages.

Phytochemicals from plants have garnered much consideration due to their high bioactive properties such as anticancer, antibacterial, and antioxidant activities. The food, pharmaceutical, and cosmetic trades still encounter main challenges in efficiently and sustainably extracting these precious molecules. Conventional extraction methods have developed over the years, and novel methods have arisen that improve environmental sustainability, effectiveness, and selectivity. This investigation deliberates the mechanics, efficacy, and application of both conventional and innovative phytochemical extraction procedures. One of the main steps towards improved bioavailability and reduced environmental footprint is the transition from solvent‐based approaches to novel methods such as supercritical fluid extraction, ultrasonic and microwave‐assisted extraction, and nanoencapsulation. Various extraction methods are depicted in Figure 2.

### 4.1 Conventional Methods

Due to their uncomplicatedness and efficacy, classical phytochemical extraction methods have been working to a greater degree. Steam distillation and solvent extraction endure as the most prevalent among them.

#### 4.1.1 Solvent Extraction

The usage of organic solvents such as acetone, methanol, and ethanol to solubilize bioactive composites from plant material is discussed as solvent extraction. Since it competently isolates an enormous range of phytochemicals, the method is often used (Laboukhi‐Khorsi et al. 2017). Greener replacements are under investigation, in the meantime, due to anxieties over solvent poisonousness, residue pollution, and environmental pollution (Chemat et al. 2019).

#### 4.1.2 Steam Distillation

Additional conventional procedure primarily used for extraction of essential oil is steam distillation. Almeida‐Couto et al. (2022) define steam distillation as a procedure whereby steam is accompanied through plant materials which prompt volatile chemicals to vaporize and condense as liquids. Although steam distillation is a very operative procedure, it inclines to create the thermolabile chemicals degrade, which would consequence in reduced yields of extraction as well as bioactivity (Chemat et al. 2020).

### 4.2 Innovative Techniques

Augmented yields with the least degradation of bioactive composites are assured by more maintainable, efficient, and selective procedures enabled by developments in extraction technologies.

#### 4.2.1 Supercritical Fluid Extraction

Supercritical carbon dioxide (SC‐CO_2_) is employed as a solvent for supercritical fluid extraction (SFE), which isolates phytochemicals under well‐ordered pressure and temperature. High choosiness, eco‐friendliness, and negligible residue of the solvent are all confirmed by this method (Yıldırım et al. 2024). For lipophilic composites such as carotenoids, flavonoids, and essential oils, SFE is mainly useful (Šojić et al. 2022). Yet, its widespread solicitation is limited by the high cost of the procedure and the requirement for specialist apparatus (de Aguiar et al. 2023).

#### 4.2.2 Ultrasound and Microwave‐Assisted Methods

With less time, solvent, and energy, ultrasound‐assisted extraction (UAE) and microwave‐assisted extraction (MAE) improve the extraction procedure. To improve mass transfer and the solubilization of bioactive composites, UAE applies high‐frequency sound waves to abolish plant cell walls (Pogorzelska‐Nowicka et al. 2024). Associated with this, MAE accelerates composite diffusion by heating the solvent and plant matrix with microwave radiation (López‐Salazar et al. 2023). Due to their effectiveness and assistance to the environment, such methods are gaining popularity progressively (Mazumder et al. 2023).

#### 4.2.3 Nanoencapsulation for Enhanced Bioavailability

Nanoencapsulation denotes to the encapsulation of phytochemicals in nanostructured carriers such as liposomes, polymeric nanoparticles, and solid lipid nanoparticles. This procedure improves the bioavailability of bioactive composites by improving their constancy, solubility, and controlled release (McClements and Öztürk 2021). Current progressions highlight the targeted delivery of phytochemicals by nanoencapsulation in both the food and pharmaceutical sectors (Guía‐García et al. 2022). Moreover, investigation identifies its capability to prolong shelf life by shielding sensitive resources from deprivation (Domínguez et al. 2021) (Table 2).

**TABLE 2:** Comparison of conventional and innovative phytochemical extraction techniques: Principles, solvents, advantages, and limitations.

Extraction method	Principle	Advantages	Limitations	Scalability and industrial readiness	Cost‐effectiveness	Sustainability and greenness	Regulatory acceptance and challenges	References
Solvent extraction	Dissolution of compounds in a liquid solvent	Simple, well‐understood, high efficiency	Solvent toxicity, environmental pollution, high energy for solvent removal	**High (Industrial)** Mature technology, widely used	**Low** Low capital cost, but high operational cost (solvent, energy)	**Poor** Hazardous solvent waste, high E‐factor	**High** Well‐established but under scrutiny for solvent residues	Laboukhi‐Khorsi et al. (2017); Chemat et al. (2019)
Steam distillation	Vaporization and condensation of volatile compounds	Solvent‐free, simple equipment	High temperature degrades thermolabile compounds, energy‐intensive	**High (Industrial)** Standard for essential oils	**Medium** Low solvent cost, but high energy cost	**Good** Uses water, but high energy demand lowers score	**High** GRAS status for essential oils	Chemat et al. (2019)
Supercritical fluid extraction (SFE‐CO_2_)	Uses CO_2_ above critical point to dissolve compounds	Solvent‐free, high purity, tunable selectivity	Very high capital cost, high operating pressure	**Medium** Established for high‐value products (e.g., hops, caffeine)	**Low** Highest capital investment, but lower running costs	**Excellent** Uses non‐toxic CO_2_ (often from waste streams), but energy‐intensive	**High** CO_2_ is GRAS; accepted for food and pharma	de Aguiar et al. (2023); More et al. (2022)
Ultrasound‐assisted extraction (UAE)	Cavitation disrupts cell walls to enhance mass transfer	Faster, reduced solvent/temperature, higher yield	Possible degradation from cavitation, scaling up can be challenging	**Medium‐High** Increasingly used in food and nutraceutical industries	**Medium** Moderate capital cost, reduces solvent consumption	**Good** Reduces solvent and energy use	**High** Considered a green and compliant technology	Chemat et al. (2020); Pattnaik et al. (2021)
Microwave‐assisted extraction (MAE)	Microwave energy heats solvents and plant matrix internally	Rapid, highly efficient, reduced solvent use	Non‐uniform heating, requires polar solvents, specialized equipment	**Medium** Growing adoption for specific bioactive compounds	**Medium** Higher capital cost than conventional, but operational savings	**Good** Reduces extraction time and solvent consumption significantly	**High** Well‐regarded; no major regulatory hurdles	López‐Salazar et al. (2023)
Pulsed electric field (PEF)	Short HV pulses electroporate cell membranes	Non‐thermal, energy‐efficient, preserves bioactivity	Limited to moist matrices, limited depth of penetration, pre‐treatment step	**Low‐Medium** Primarily pre‐treatment; emerging for liquid foods	**Medium** Efficacy can reduce downstream costs, but capital is high	**Excellent** Very low energy per kg processed, no solvents	**Medium** Approved for food processing; acceptance as extraction aid growing	Ferraz and Silva (2024); Meijer et al. (2021)
Pressurized liquid extraction (PLE)	High temp/pressure to enhance solubility and kinetics	High efficiency, automated, low solvent consumption	High equipment cost, high temperature may degrade compounds	**Medium** Used for analytical standards and high‐value extracts	**Medium‐High** High capital and maintenance costs	**Good** Reduced solvent use offsets high energy input	**High** Accepted technique, especially in analytics	Madia et al. (2021)
Enzyme‐assisted extraction (EAE)	Enzymes break down cell walls to release bound compounds	Mild conditions, highly selective, improves yield	Cost of enzymes, requires optimization of pH/temp, longer time	**Low‐Medium** Used in specific sectors (e.g., juice, oil); cost is barrier	**Low** Enzymes are expensive, making process cost high	**Excellent** Highly specific, works with water, minimal energy	**Medium** Enzymes must be GRAS; process‐specific approval	Raj et al. (2024); More et al. (2022)
Deep eutectic solvent extraction (DES)	Uses natural eutectic mixtures as designer solvents	Tunable, biodegradable, high solubility for polar/non‐polar compounds	High viscosity limits mass transfer, purification challenges, nascent tech	**Low (Lab Scale)** Significant research needed for scaling and recycling	**Low‐Medium** Solvents are cheap, but recovery/reuse is key for cost	**Excellent** Typically made from natural, biodegradable compounds	**Low** No specific framework; novel solvents require full safety assessment	Saini et al. (2022); Meijer et al. (2021)
Ionic liquid extraction	Uses ionic liquids as solvents with high solvation power	High thermal stability, negligible vapor pressure, tunable	High cost, potential toxicity, complex synthesis and recycling	**Low (Lab Scale)** Toxicity and cost are major barriers to scale‐up	**Low** Most expensive solvent option	**Poor** Green due to no volatility, but poor biodegradability and eco‐toxicity	**Low** Significant regulatory hurdles due to unknown long‐term toxicity	Bas (2024); Chemat et al. (2019)
Nanoencapsulation	Encapsulation of bioactives in nanoscale carriers	Enhances stability, bioavailability, and controlled release	Complex formulation, high cost, characterization challenges	**Medium** Growing in nutraceutical and functional food sectors	**Low** High processing and material costs	**Medium** Can reduce waste but relies on energy‐intensive processes	**Medium** Evolving regulations for nanomaterials in food (nano‐specific)	Bazana et al. (2019); McClements and Öztürk (2021); Pavithra and Manimaran (2024)

Abbreviations: GRAS, generally recognized as safe; HV, high voltage.

Phytochemical extraction has revolutionized and shifted from conventional solvent‐based approaches to more effectual and environmentally friendly developing technologies. Nanoencapsulation, ultrasonic and microwave‐based approaches, and supercritical fluid extraction all offer auspicious means to improve extraction yields, selectivity, and bioavailability. The necessity for substitute extraction approaches that exploit phytochemical recovery with negligible environmental footprint is also driven by the rising focus on green chemistry and sustainable procedures. To incorporate these technologies into industrial production without compromising on their effectiveness and cost‐effectiveness, forthcoming investigation must focus on augmenting them to suit large‐scale applications. Through the usage of these advancements, industries can leverage phytochemicals to the completest, making their solicitations better in pharmaceuticals, nutraceuticals, and functional foods.

## 5 Phytochemicals in Functional Food Development

Plants harbor bioactive composites known as phytochemicals with numerous health benefits. Due to their health elevation and disease prevention competences, the addition of phytochemicals to functional foods has produced much interest. The purpose of functional food production is to augment health effects through the fortification of staple foodstuffs to improve their nutrient content. This is an approach that incorporates bioactive composites in usual foods such as beverages, snacks, and milk substitutes. In addition, phytochemicals are serious for the prevention of metabolic disorders such as diabetes and obesity, the elevation of cognitive function, the upkeep of gut microflora, and cardiovascular health. This investigation explores methods of fortification and the precise health welfares of phytochemicals in functional food fabrication.

### 5.1 Fortification Strategies

One of the key approaches for refining public health is the fortification of simple meals with phytochemicals. In this procedure, broadly used cereals such as rice, wheat, and maize are fortified with bioactive composites such as polyphenols, flavonoids, carotenoids, and other composites derived from plants. As per Aguilar‐Pérez et al. (2023), nano‐fortification procedures improve these chemicals' bioavailability, certifying better absorption and efficacy. Whereas developing technologies explore the application of encapsulation in defending such compounds from collapse, conventional fortification approaches entail the incorporation of phytochemicals in bread and dairy foods. Beyond the ornamental nutrient content of the staple foods, fortification decreases the threat of chronic ailments and delivers health anticipation benefits. Over the past few years, the petition for value‐added functional food products fortified with phytochemicals has been rising rapidly. The most prevalent categories for creating functional foods are beverages, snacks, and dairy substitutes. Functional beverages derived from cereal grains like barley and oats comprise high levels of bioactive phytochemicals that provision health, investigated by Xiong et al. (2022). In addition, beverages derived from fruit such as those derived from cherries and blueberries have antidiabetic and anti‐inflammatory properties (Gonçalves et al. 2022). Almond milk and soy milk, two dairy replacements fortified with plant bioactives, meet the demands of customers seeking plant health benefits without lactose intolerance problems. To ensure that the phytochemicals are conserved in their strength and that supreme health benefits for the customers are attained, products are advanced using optimized processing circumstances.

## 6 Targeted Health Benefits

### 6.1 Cardiovascular Health

**FIGURE 3:**
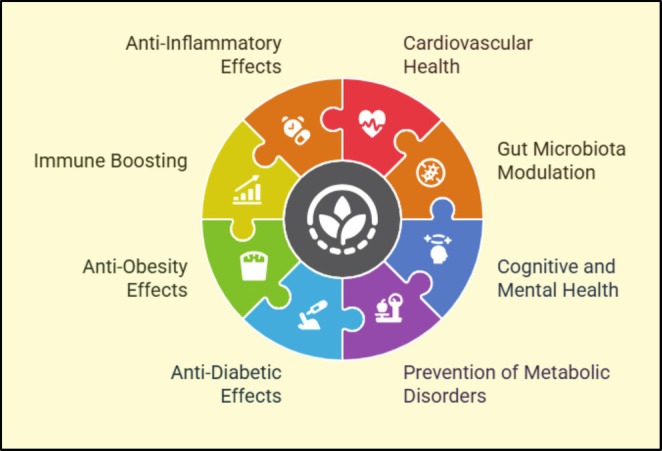
Numerous health benefits that are connected to the various phytochemicals that can be seen in plant‐based diets. These phytochemicals have a substantial role in the prevention of diseases; for instance, cancer, cardiovascular disease, diabetes, neurodegenerative and inflammatory diseases, and metabolic disorders.

Phytochemicals improve blood flow, decrease cholesterol, and lessen oxidative stress, so these are vital for cardiovascular well‐being. Based on investigation, the flavonoids and polyphenols in functional foods such as dark chocolate and berries have cardioprotective effects (Centner et al. 2023). These composites contain anti‐inflammatory and antioxidant effects that defend the body against high blood pressure and atherosclerosis. In addition, it has been revealed that plant sterols and stanols, which are present in fortified margarine and yogurt, have the capability to meaningfully decrease LDL cholesterol levels (Bule et al. 2020). Through the development of endothelial function and modulation of lipid metabolism, functional meals augmented with phytochemicals decrease the prevalence of cardiovascular disease and promote cardiovascular health (Centner et al. 2023). Various health benefits have been explored in Figure 3.

### 6.2 Gut Microbiota Modulation

The human gut flora distresses the immune system, digestion, and general health (Shi et al. 2023). The gut microbiota conformation is controlled by phytochemicals that overpower harmful pathogens and endorse the proliferation of beneficial bacteria. Sudheer et al. (2022) clarify that plant dietary fibers, polyphenols, and prebiotics augment gut microbial variety and enhance digestion while dropping inflammation. Yogurt and kimchi represent some of the fermented functional foods that comprise bioactive composites accomplished of stimulating probiotic bacteria proliferation in the gut (Sudheer et al. 2022). Also, green tea and citrus fruits' phytochemicals have antibacterial physiognomies that preserve homeostasis within the gut while shielding against disorders in the digestion process (Sudheer et al. 2022).

### 6.3 Cognitive and Mental Health

In the fabrication of functional foods there has been augmented interest in the neuroprotective properties of phytochemicals. Improved mental wellness and cognitive functioning have been associated with flavonoids, predominantly those found in tea, cocoa, and berries (Melrose 2023). Improved mental wellness and cognitive functioning have been associated with flavonoid intake in human clinical studies. For example, a cross‐sectional study demonstrated that higher dietary intake of flavonoids correlates with enhanced cognitive performance in older adults. Interventions with cocoa flavanols (500–900 mg/day) over 8–12 weeks have shown significant improvements in cognitive functions such as attention, processing speed, and working memory in healthy adults and those with mild cognitive impairment (Yang et al. 2021). These composites decrease the risk of neurodegenerative diseases such as Parkinson's and Alzheimer's due to anti‐inflammatory and antioxidant effects. One of the key methods that dietary polyphenols affect mood, memory, and cognitive function, says Jun Liu et al. (2023), is via the gut‐brain axis. Polyphenols modulate gut microbiota composition, increasing beneficial bacteria like Bifidobacterium and Lactobacillus, which produce neuroactive metabolites such as short‐chain fatty acids (SCFAs) that influence brain health, reported by Berding et al. (2021). Naomi et al. (2023) reported that human studies have shown that polyphenol‐rich diets can reduce systemic inflammation and oxidative stress, which are linked to cognitive decline. Dark chocolate and turmeric pervaded drinks are a couple of examples of functional foods added with such phytochemicals that have shown effectiveness in boosting brain functioning and plummeting feelings of depression and anxiety feelings (Jun Liu et al. 2023). For example, dark chocolate consumption (with high flavonoid content) was associated with improved mood and cognitive outcomes in adults (Yang et al. 2021), while curcumin (from turmeric) reduced anxiety and depression scores in clinical settings (Naomi et al. 2023).

The effectiveness and acceptance of strategies for incorporating neuroprotective phytochemicals into functional foods vary significantly. Fortification involves adding purified phytochemicals (e.g., polyphenols, vitamins) to food products and is widely used due to its scalability and precise dosing. However, its effectiveness is limited by bioavailability issues and potential sensory changes in food products. For example, iron fortification compounds often face challenges with oxidative stability and consumer acceptance due to off‐flavors (Kaur et al. 2022). Fermentation leverages microbial processes to enhance the bioavailability and bioactivity of phytochemicals. This strategy is increasingly accepted for its ability to improve nutrient absorption and generate beneficial metabolites that support gut‐brain axis communication. Human studies have shown that fermented foods (e.g., kefir, fermented vegetables) can positively modulate gut microbiota and reduce inflammation, thereby indirectly supporting cognitive health (Appleton 2018). Encapsulation (e.g., nano emulsions, liposomes) protects phytochemicals from degradation during processing and digestion, thereby enhancing their stability and bioavailability. Naomi et al. (2023) reported that encapsulated curcumin has shown improved absorption and neuroprotective effects in human trials compared to non‐encapsulated forms. However, encapsulation is cost‐intensive and may face regulatory hurdles. Among these strategies, food matrix incorporation (e.g., whole food‐based approaches like adding berry extracts or turmeric directly into foods) is often more effective than fortification with isolated compounds because it preserves the synergistic effects of multiple phytochemicals and fiber, which enhance bioavailability and physiological benefits. Human clinical data suggest that whole‐food approaches (e.g., blueberry supplementation) yield more consistent cognitive benefits compared to isolated compounds due to their broader action on inflammation, oxidative stress, and gut microbiota (Lamport et al. 2012).

### 6.4 Prevention of Metabolic Disorders

Metabolic disorders and obesity comprising diabetes are concerning public health problems. By regulating glucose metabolism, accumulative insulin sensitivity, and plummeting adipogenesis, phytochemicals have been extremely potent in the treatment of abundant diseases (Saad et al. 2017). It has been found that polyphenols in blueberries, cinnamon, and green tea improve the metabolic process and lessen blood sugar levels (Martemucci et al. 2024). Citrus flavonoids also control weight by dropping inflammation and changing lipid metabolism. A natural and effective way of handling and stopping metabolic disorders, functional food products augmented with these bioactive agents improve overall health consequences (Table 3).

**TABLE 3:** Health benefit, phytochemicals involved, mechanism of action, food sources.

Health benefit	Phytochemicals involved	Mechanism of action	Food sources	References
Cardiovascular health	Flavonoids, polyphenols, carotenoids, anthocyanins	Antioxidant activity, reduction of LDL oxidation, anti‐inflammatory effects, improved endothelial function, and blood pressure regulation	Berries, citrus fruits, nuts, green tea, dark chocolate, whole grains	Aguilar‐Pérez et al. (2023); Bule et al. (2020); Granato et al. (2020); Centner et al. (2023)
Gut microbiota modulation	Polyphenols, dietary fibers, lignans, prebiotics	Modulation of gut microbial composition, enhancement of beneficial bacteria (e.g., Lactobacillus, Bifidobacterium), production of short‐chain fatty acids (SCFAs), and reduction of pathogenic bacteria	Whole grains, legumes, fruits (e.g., apples, berries), vegetables, fermented foods	Dingeo et al. (2020); Sudheer et al. (2022); Shi et al. (2023); Mirmohammadali and Rosenkranz (2023)
Cognitive and mental health	Flavonoids, curcumin, resveratrol, catechins, omega‐3 fatty acids	Anti‐inflammatory and antioxidant effects, reduction of neuroinflammation, promotion of neurogenesis, and modulation of gut‐brain axis	Blueberries, walnuts, turmeric, green tea, fatty fish, dark chocolate	Kim et al. (2024); Liu et al. (2023); Bakoyiannis et al. (2019); Howes et al. (2020)
Prevention of metabolic disorders	Polyphenols, flavonoids, alkaloids, terpenoids, saponins	Regulation of glucose metabolism, improvement of insulin sensitivity, reduction of adipogenesis, and modulation of lipid metabolism	Citrus fruits, berries, green tea, whole grains, legumes, nuts	Saad et al. (2017); Gandhi et al. (2020); Martemucci et al. (2024); Nainu et al. (2023)
Anti‐diabetic effects	Flavonoids, phenolic acids, alkaloids, saponins	Inhibition of carbohydrate‐digesting enzymes, enhancement of insulin secretion, improvement of glucose uptake, and reduction of oxidative stress	Bitter melon, fenugreek, cinnamon, turmeric, green tea, berries	Saad et al. (2017); Gandhi et al. (2020); Martemucci et al. (2024)
Anti‐obesity effects	Catechins, capsaicin, curcumin, resveratrol, dietary fibers	Suppression of appetite, enhancement of fat oxidation, reduction of adipogenesis, and modulation of gut microbiota	Green tea, chili peppers, turmeric, grapes, whole grains, legumes	Saad et al. (2017); Martemucci et al. (2024); Nainu et al. (2023)
Immune boosting	Polyphenols, flavonoids, carotenoids, vitamin C, zinc	Enhancement of immune cell function, reduction of oxidative stress, and anti‐inflammatory effects	Citrus fruits, berries, carrots, spinach, nuts, seeds	Gonçalves et al. (2022); Vlaicu et al. (2023); Ashique et al. (2024)
Neuroprotection	Curcumin, resveratrol, flavonoids, omega‐3 fatty acids	Reduction of neuroinflammation, inhibition of amyloid‐beta aggregation, promotion of synaptic plasticity, and antioxidant effects	Turmeric, grapes, walnuts, fatty fish, green tea	Kim et al. (2024); Melrose (2023); Howes et al. (2020)
Anti‐inflammatory effects	Curcumin, resveratrol, quercetin, catechins, omega‐3 fatty acids	Inhibition of pro‐inflammatory cytokines (e.g., TNF‐α, IL‐6), reduction of oxidative stress, and modulation of inflammatory pathways	Turmeric, grapes, green tea, fatty fish, berries	Forni et al. (2019); He et al. (2023); Nainu et al. (2023)
Antioxidant effects	Polyphenols, flavonoids, carotenoids, vitamin C, vitamin E	Scavenging of free radicals, reduction of oxidative stress, and protection of cellular components from damage	Berries, citrus fruits, nuts, seeds, green leafy vegetables	Al‐Gubory (2018); Forni et al. (2019); He et al. (2023)
Cancer prevention	Curcumin, resveratrol, catechins, sulforaphane, lycopene	Induction of apoptosis in cancer cells, inhibition of angiogenesis, and reduction of oxidative stress and inflammation	Turmeric, grapes, green tea, broccoli, tomatoes	Bule et al. (2020); Granato et al. (2020); He et al. (2023)
Liver health	Silymarin, curcumin, catechins, resveratrol	Reduction of oxidative stress, anti‐inflammatory effects, and promotion of liver detoxification	Milk thistle, turmeric, green tea, grapes	Bule et al. (2020); He et al. (2023); Nainu et al. (2023)
Bone health	Isoflavones, lignans, polyphenols, vitamin K	Enhancement of bone mineral density, reduction of bone resorption, and promotion of osteoblast activity	Soybeans, flaxseeds, green leafy vegetables, nuts	Bule et al. (2020); Granato et al. (2020)
Skin health	Polyphenols, carotenoids, vitamin C, vitamin E	Protection against UV‐induced damage, reduction of oxidative stress, and promotion of collagen synthesis	Citrus fruits, carrots, tomatoes, nuts, seeds	Bule et al. (2020); Granato et al. (2020)
Eye health	Carotenoids (lutein, zeaxanthin), anthocyanins, vitamin A	Protection against oxidative damage, reduction of age‐related macular degeneration, and improvement of visual acuity	Carrots, spinach, kale, berries, eggs	Bule et al. (2020); Granato et al. (2020)
Anti‐aging	Resveratrol, curcumin, polyphenols, omega‐3 fatty acids	Reduction of oxidative stress, inhibition of cellular senescence, and promotion of mitochondrial function	Grapes, turmeric, green tea, fatty fish, nuts	Forni et al. (2019); He et al. (2023); Melrose (2023)
Anti‐microbial effects	Alkaloids, flavonoids, phenolic acids, terpenoids	Inhibition of bacterial and viral growth, enhancement of immune response, and reduction of microbial adhesion	Garlic, ginger, turmeric, green tea, berries	Bule et al. (2020); Granato et al. (2020); Nainu et al. (2023)
Anti‐allergic effects	Quercetin, catechins, curcumin, resveratrol	Inhibition of histamine release, reduction of inflammatory mediators, and modulation of immune response	Apples, green tea, turmeric, grapes, onions	Bule et al. (2020); Granato et al. (2020); Nainu et al. (2023)
Wound healing	Curcumin, polyphenols, vitamin C, zinc	Promotion of collagen synthesis, reduction of inflammation, and enhancement of tissue repair	Turmeric, citrus fruits, nuts, seeds	Bule et al. (2020); Granato et al. (2020)
Anti‐hypertensive effects	Flavonoids, polyphenols, omega‐3 fatty acids	Vasodilation, reduction of oxidative stress, and inhibition of angiotensin‐converting enzyme (ACE)	Berries, green tea, fatty fish, dark chocolate	Centner et al. (2023); Martemucci et al. (2024)

### 6.5 Anti‐Inflammatory and Antioxidant Properties

Several diseases comprising cancer, arthritis, and cardiovascular diseases are interrelated to chronic inflammation. Potent anti‐inflammatory and antioxidant properties found in phytochemicals help fight oxidative stress and the alleviation of inflammation. Forni et al. (2019) stated that fruits, vegetables, and herbs have polyphenols and flavonoids that scavenge free radicals and avert cellular damage. Functional foods comprising very strong anti‐inflammatory competences have been shown to be green tea, ginger comprising foodstuffs, and foods with a flavor of turmeric. Consistent consumption of such meals is shown to improve overall well‐being and assist in reducing the likelihood of getting chronic diseases.

One of the promising tactics to enlighten public health and avert chronic diseases is the addition of phytochemicals to functional diets. The nutritional excellence of staple foods is improved by fortification approaches, while innovative functional food products cater to customers seeking plant‐based health benefits. Cardiovascular defense, regulation of gut microbiota, cognitive improvement, deterrence of metabolic disorders, and anti‐inflammatory effects are certain of the targeted health benefits. Progressive processing techniques will progressively improve the bioavailability and potency of phytochemicals as investigation continues, paving the way for a healthier prospect. Functional foods can revolutionize nutrition and subsidize to long‐term well‐being by bringing these bioactive composites into daily meals.

## 7 Sustainability and Phytochemical Utilization

To convert agricultural deposit into valuable constituents, food waste valorization plays an important role in sustainable development. One potential process of enhancing food security, reducing environmental burden, and developing circular economy methods is the bioactive compound retrieval from food wastes, including grape seeds and orange peels (Sorrenti et al. 2023). These bioactives, comprising important health benefits and potential applications in the pharmaceutical, cosmetic, and food sectors, are signified by polyphenols, carotenoids, and flavonoids (Fidelis et al. 2019). Nanoencapsulation of these bioactives has been shown to enhance their absorption and bioavailability. For instance, nanoencapsulated curcumin demonstrates up to 90% absorption efficiency compared to 20%–30% for non‐encapsulated forms, while nanoemulsions of essential oils improve bioavailability by 50%–300% depending on the formulation and particle size (Onoue et al. 2014). One of the methods to cut down on waste and improve the usage of resources in a sustainable manner is through extracting bioactive elements from food wastage. Flavonoids and essential oils, having antibacterial and antioxidant actions, are present in large quantities in citrus peels (Maqbool et al. 2023). Green substitutes for conventional solvent‐based extraction procedures are offered by high‐tech extraction procedures such as supercritical fluid extraction and ultrasound‐assisted extraction (More et al. 2022). However, safety concerns regarding nanoencapsulation must be addressed. Some nanoparticles may induce oxidative stress, inflammation, or cytotoxicity depending on their physicochemical properties (e.g., size, charge, and composition). Cationic nanoparticles can cause greater cellular toxicity compared to anionic or neutral ones, and metal‐based nanoparticles like silver or zinc oxide may accumulate in tissues, leading to long‐term toxicity (Haripriyaa and Suthindhiran 2023). Yet another noteworthy source of polyphenols, which have been associated with benefits for metabolic and cardiovascular health, is grape seeds (Ben‐Othman et al. 2020). By adding value to these by‐products and increasing human health, as well as inspiring a circular economy, value adding can lead to the advancement of functional foods and nutraceuticals (Seyyedi‐Mansour et al. 2024). Food waste valorization has majorly positive influences on the environment and the economy. Landfill waste, carbon footprints, and novel revenue streams can be reduced by industries by exploiting agricultural by‐products in substitute usages (Berenguer et al. 2022). Sustainable waste management and innovation in extracting bioactive composites are highly reinforced by government inducements and policies (Yadav et al. 2024). The amalgamation of bioactives into food products also improves nutritional content and shelf life. Polyphenols from fruit peels have been found to upsurge food functionality and preservation and act as a natural substitute for synthetic preservatives (Nirmal et al. 2023). Even though bioactive extraction technology is emerging in a promising direction, trepidations regarding cost‐effectiveness and scalability remain. To proficiently optimize extraction approaches and integrate them into industrial procedures, further investigation and development are required (Panzella et al. 2020).

Green extraction technologies hold promise for food waste recycling and reuse, yet there are prohibitive, usually unspoken, barriers to their large‐scale industrial use beyond their underlying technology viability. Small and medium‐sized businesses (SMEs) are unable to handle the capital expense necessary for sophisticated technology such as supercritical CO_2_ extraction. As a result, they are subsequently forced to employ less sustainable conventional methods that are cheaper (More et al. 2022; Onipede et al. 2024). The already difficult regulatory environment has deteriorated further for the economy. It is not known how new food ingredients derived from waste streams, particularly those derived from new solvents (e.g., nanoencapsulation) or process conditions (e.g., ionic liquids, deep eutectic solvents), will be licensed. It follows a long and expensive journey to market because of the large safety data requirements of regulatory agencies, suppressing innovation (Bahorun et al. 2019; Meijer et al. 2021). Küster‐Boluda and Vidal‐Capilla (2017) and Nystrand and Olsen (2020) both show that customer acceptance cannot be guaranteed and that “waste‐derived” ingredients and “processed” functional meals are likely to raise doubts and consequently commercial failure. The commercial valorization of citrus waste represents an excellent example of a case study that is able to overcome those obstacles. Companies have utilized microwave‐ and ultrasound‐assisted extraction to yield flavonoids and polyphenols, which are used in beverages and dietary supplements. This is a sustainable circular economy (Maqbool et al. 2023; Liu et al. 2024). To allow for a genuine circular phytochemical economy, these policy and industrial hurdles need to be overcome. Legislators need to develop transparent and complementary frameworks for waste‐to‐value products; businesses need to invest in pilot‐scale facilities and cost‐sharing; and researchers need to emphasize techno‐economic studies to establish commercial viability (Berenguer et al. 2022; Ospina‐Maldonado et al. 2024).

### 7.1 Sustainable Agriculture

The goal of sustainable agriculture is to preserve ecological equilibrium and put natural resources to effective usage. To improve crop resistance, management of pests and climate change adaptation, phytochemicals play a vital role. Exhausting them as growth promoters and biopesticides endorses eco‐friendly farming practices and decreases chemical input dependence (Espinosa‐García 2022). Nano formulations of phytochemicals, such as nano emulsions of neem oil, have shown improved efficacy, requiring 50% lower doses compared to conventional formulations for effective pest control (Malik and Waheed 2023). Powerful insecticidal and fungicidal activities concentrate phytochemicals from plants as an effective substitute for synthetic pesticides (Divekar et al. 2022). The effectiveness of plant‐derived essential oils from neem and citrus peels in monitoring pest organisms in agriculture and upholding soil health has been studied in excessive detail (Roberts and Mattoo 2018). Terpenoids and alkaloids are some examples of secondary metabolites that are organic protective agents against infections and insect herbivores. The environmental impact of modern agriculture can be meaningfully abridged by integrating them into integrated pest management (IPM) methods (Sharma 2024). However, the environmental fate and potential toxicity of these nano formulations must be considered. Studies indicate that metal‐based nanoparticles (e.g., copper or silver) used in agriculture may accumulate in soil, affecting microbial diversity and soil health (Malik and Waheed 2023). The benefits of phytochemicals for soil fertility are also demonstrated by studies. Plant biofertilizers from waste products, for example, improve nutrient availability and microbial variety in the soil, which recovers crop yield in general (El‐Ramady et al. 2022).

### 7.2 Role in Plant Resilience and Adaptation to Climate Change

Abiotic stresses elicited by global warming such as drought and temperature variations influence crop yield and quality. Phytochemicals play a noteworthy role in plant stress responses, which augment adaptability and resistance (Vaughan et al. 2018). Nanoencapsulation of stress‐mediated phytochemicals, such as antioxidants, can enhance their stability and efficacy. For example, nanoencapsulated ascorbic acid applied to crops under drought conditions improved antioxidant activity by 40% compared to non‐encapsulated forms (Pautler and Brenner 2010). Based on the newest study, stress‐mediated phytochemical accumulation improves antioxidant activity in plants and augments their resistance to adverse circumstances (Ullah et al. 2024). Climate‐resistant crops with improved nutritional and therapeutic properties can be developed through the exploitation of these adaptation mechanisms (Mashabela et al. 2022). Pharmacokinetic studies in plants show that nanoencapsulated phytochemicals have longer retention times and improved systemic distribution, ensuring sustained release and better stress protection (Haleem et al. 2023). Moreover, biodiversity and long‐term food security are certified through the fabrication of traditional and stress‐resistant crop diversities. Ancient crop varieties have been found to own exceptional phytochemicals that permit sustainable agricultural practices (Berni et al. 2018).

## 8 Circular Food Economy and Challenges in Phytochemical Integration

**FIGURE 4:**
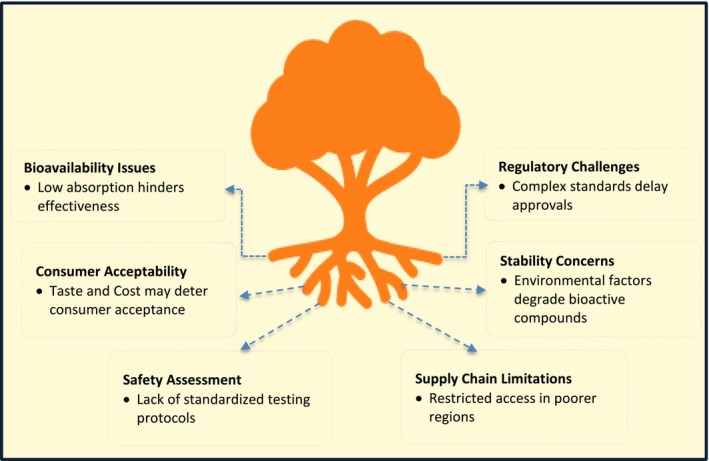
Key challenges of phytochemical applications in the food system have been highlighted through the fascinating importance of bioavailability and stability that enhance the efficacy of biologically active phytochemicals. While safety and regulatory concerns are necessary for assuring user protection, along with adherence to food standards, the success of phytochemical‐enriched food products depends on consumer acceptability, as consumer indulgence and views can have a substantial impact on demand.

The globular food economy purposes to integrate sustainable tactics for waste and food production. The solicitation of this model in terms of phytochemicals certifies negligible waste generation and optimum resource operation (Matías et al. 2024). Transformation of agricultural remains into high‐value products is a dominant element in a zero‐waste food production system. Pharmaceuticals, cosmetics, and functional foods can all be subjected to the bioactive composites that are isolated from fruit and vegetable leftovers (Fidelis et al. 2019). Plastic pollution has abridged due to biotechnology novelties that have simplified the process of altering food waste into biodegradable packing materials (Srivastava et al. 2024). These inventions demonstrate how phytochemical valorizations can contribute to founding a waste‐free, sustainable economy. Policy systems and customer consciousness are dynamic in permitting the transition to a circular food economy. Invention and industry participation are reinvigorated through incentives for waste administration and sustainable food fabrication (Nawaz et al. 2024). Furthermore, investigation on the application of nanotechnology in phytochemical application has opened up avenues for the sustainable fabrication of products (Mohapatra et al. 2023). Phytochemical‐infused nanocomposite packing food has a prolonged shelf life and includes fewer artificial additives (Patel et al. 2020). Eventually, cooperation among scholars, policymakers, and industry stakeholders is integral to the successful application of a circular food system. Food systems can become sturdier, more maintainable, and efficient by adopting phytochemicals into a zero‐waste agenda (Ulian et al. 2020) (Figure 4). Despite the potential, challenges include consumer acceptance, regulatory hurdles, and bioavailability issues. For example, nanoencapsulation systems must undergo rigorous safety assessments to ensure they do not pose health risks. Regulatory agencies like the FDA and EMA require comprehensive toxicity and pharmacokinetic data for approval (Onoue et al. 2014; Haripriyaa and Suthindhiran 2023).

Phytochemicals hold vast prospects in most fields, chiefly in food, medicine, and agriculture. There are abundant barriers to their inclusion in everyday usages; though there is concern regarding suitability by the customer, safety and regulatory challenges, and bioavailability and steadiness, which are noteworthy barriers. To be efficiently utilized in products that are sustainable and healthy, refining these issues is required to be overcome (Eissa et al. 2023).

### 8.1 Bioavailability and Stability

The bioavailability and constancy of phytochemicals are one of the key impediments to their application. The therapeutic effectiveness of most phytochemicals is hindered by their inability to be readily absorbed and their low solubility within the human entity. As an example, environmental aspects such as heat, light, and exposure to oxygen may damage polyphenols, flavonoids, and carotenoids swiftly (Srivastava et al. 2024). Nanotechnology‐based delivery systems such as lipid‐based carriers and nanoencapsulation have been discovered to improve the constancy and bioavailability of phytochemicals. Phytochemicals may be encapsulated in biodegradable polymers to improve their release profile and uphold stability against dilapidation (Mehta et al. 2024). Moreover, it has been found that the usage of micellar systems and emulsions increases the aqueous solubility of hydrophobic phytochemicals, thereby enhancing their bioavailability (Alfei et al. 2021). Phytochemical permanence is further prejudiced by food processing and storage circumstances. Evidence has shown that conservation of bioactive chemicals is meaningfully abridged through high‐temperature processing procedures like baking and frying (Langston et al. 2023). Hence, the integrity of phytochemicals must be conserved with innovative packing options and optimized processing systems. The other factor manipulating phytochemical bioavailability is how phytochemicals interrelate with other food constituents. Whereas some phytochemicals undergo metabolic modifications that decrease their activity, and others require co‐factors for supreme absorption (Onipede et al. 2024). To improve absorption effectiveness, studies are ongoing on the effects of food matrix and co‐administration of garnishes such as piperine and curcumin. In addition, the effectiveness of some phytochemicals is further abridged by enzymatic degradation in the gastrointestinal tract. To evade bioactives from degrading too early, targeted delivery approaches such as enteric coating and controlled release formulations have been planned (Naik et al. 2023).

### 8.2 Regulatory and Safety Considerations

To ensure efficacy and safety, strict compliance is compulsory with regulatory standards when blending phytochemicals into foods and drugs. Standardization is challenging worldwide due to diverse legislation governing the usage of bioactive chemicals by republics (Pavithra and Manimaran 2024). The nonexistence of a complete mechanism for assessing phytochemicals' safety is one of the primary issues. Phytochemicals, unlike artificial drugs, may have complicated mixtures of ingredients that confound the assessment of their toxicological profiles and pharmacokinetics (Bahorun et al. 2019). Therefore, the development of standardized testing protocols and recognized analytical approaches is necessary for regulatory authorization. The presence of impurities and adulterants in products resulting from herbs and phytochemicals is another main concern. Stringent quality switch processes have been recognized as a direct result of critical safety issues hastened by heavy metal pollution, residues of pesticides, and microbial contamination (Zhou et al. 2019). Sophisticated analytical techniques and the usage of good manufacturing processes (GMP) can help certify the homogeneity and quality of the ended product (Dayane 2023). Phytochemical labeling of food products and entitlements of health benefits are also being examined. As a basis for any health benefit statements, regulators such as the FDA and EFSA need compelling scientific indication, which necessitates extensive clinical tests and investigation studies (Krishnaswamy 2024). For small and medium‐sized enterprises to sell goods that are phytochemical‐based, this could demonstrate to be an insurmountable barrier. Regulation adherence is compounded by the stability of phytochemicals throughout shipment and storage. The therapeutic activity and safety profile of numerous bioactive molecules are exaggerated due to their degradation over time. To meet regulatory requirements, it is vigorous to have proper storage circumstances and stability testing protocols in place (Wang et al. 2023).

### 8.3 Consumer Acceptance

Consumers often resist phytochemical‐based products even though they have health welfares due to difficulties with taste, cost, and availability. Certain phytochemicals, such as polyphenols, own an astringent or bitter flavor that can make them intolerable to many customers (Hasan et al. 2024). To improve palatability, formulation methods such as flavor masking and microencapsulation have been deliberate. Another noteworthy barrier to customer acceptance is price. Phytochemicals are expensive to extract and purify, so the product is more expensive than man‐made substitutes (Meijer et al. 2021). The target of extraction technology novelty, like enzymatic hydrolysis and supercritical fluid extraction, is to decrease production costs without compromising yields (Khademi 2024). Success in the marketplace also has much to do with the adoption and confidence of customers in phytochemical‐based products.

Most prominently, when it comes to substitute medicine and nutraceuticals, customers are still skeptical about the effectiveness and safety of natural bioactives (Abdel‐Tawab 2018). Third‐party certification, evidence‐based advertising, and open labeling can all play a role in building customer confidence. Superior phytochemical products are not always obtainable everywhere, while the functional food and dietary supplement marketplace is growing in industrialized countries; supply chain constraints frequently limit access in poorer republics (Bahorun et al. 2019). Accessibility and affordability can be improved by initiatives that endorse sustainable manufacturing procedures and local sourcing. Customer acceptance is also prejudiced by traditional eating habits and cultural insights. Although phytochemicals and herbal medicine have a long tradition in certain cultures, they are suspect in others (Naik et al. 2023). Public health enterprises and education can help bridge the gap and inspire the incorporation of phytochemicals into daily meals. Finally, chances for phytochemical integration have been enabled by personalized nutrition and the growing biohacking community. Customer demand for bioactive composites addressing specific health necessities can be evoked by developments in nutrigenomics and personalized dietary supervision (Bazana et al. 2019). Product innovation and targeted elevation will be key to overcoming customer resistance as scientific consideration of phytochemical benefits continues to recover. A lot of prospective exists for health and sustainability, with phytochemicals being added into food, medicine, and farming. Their full potential is not yet obtainable, however, due to issues of bioavailability, regulation, and receipt by clienteles. To advance past these boundaries, standardization programs, customer training, and advancements in nanotechnology will be key. Phytochemical‐based therapies distribution and being efficiently adopted depends upon continuous investigation and multidisciplinary association.

## 9 Conclusion and Future Perspectives

**FIGURE 5:**
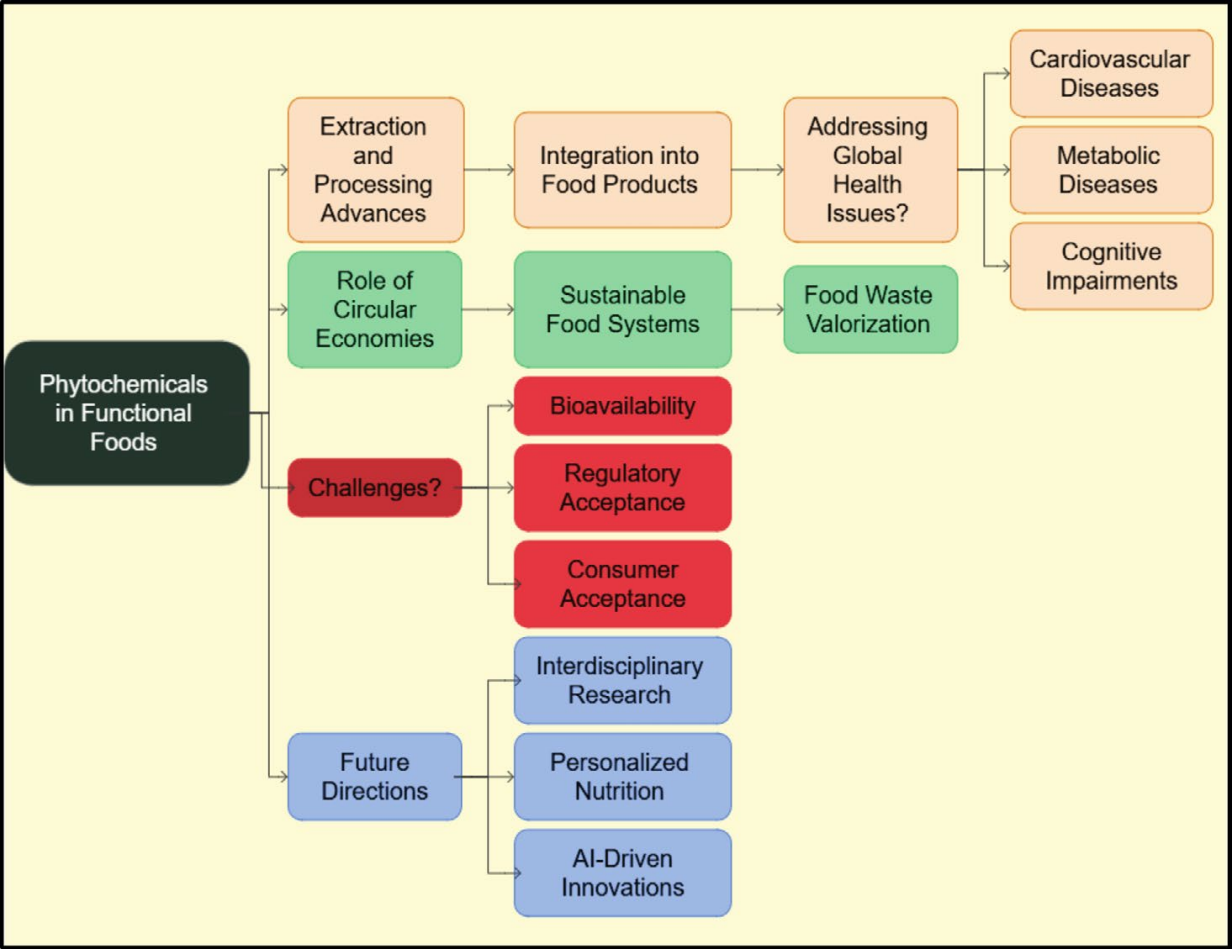
It exhibits the multifaceted approaches of this article that explore phytochemicals in functional foods; similarly, it underlines the prominence of economies in the development of sustainable practices that decrease waste. It also highlights the extraction and handling. Furthermore, it proposes future guidelines for innovations and research, besides addressing the miscellaneous challenges that arise in this field.

One of the most promising approaches to enhancing human health and endorsing sustainability in food production is the usage of phytochemicals in functional foods. Supercritical fluid extraction and nanoencapsulation are two technologies that have augmented the bioavailability and effectiveness of these bioactive complexes many times over. Phytochemical‐enriched functional foods have been found to boost metabolic and cognitive well‐being, advance gut flora, and accomplish chronic diseases. In addition, the application of phytochemicals in sustainable agriculture and food waste valorization certifies resource conservation and environmental sustainability. Widespread solicitation of functional foods supplemented with phytochemicals is delayed by several encounters despite their enormous potential. To exploit their influence, problems relating to customer perception, constancy, and regulatory adequacy need to be addressed. To improve the benefits of phytochemicals, forthcoming investigation needs to focus on formulating personalized nutritional regimens, incorporating artificial intelligence in food product expansion, and promoting interdisciplinary efforts. Functional phytochemical‐rich foods could have the competence to make great impacts on international sustainable food systems and refining public health by alleviating these problems. A summary of various approaches is concluded in Figure 5.

Harnessing technological advances and interdisciplinary associations to enhance their effectiveness and availability is central to the destiny of phytochemicals in functional food design. Tailoring functional foods based on microbiota and genomic profiles suggests a new approach to optimize individual health consequences. By predicting interactions among bioactive composites, optimizing extraction procedures, and designing tailored functional foods, machine learning and artificial intelligence will revolutionize phytochemical science. In addition, the bioavailability and steadiness of phytochemicals will be improved further by advances in nanotechnology, and their prospects for therapeutic solicitations will be augmented. Future investigations would aim at incorporating phytochemicals into circular food economies by exploiting their use in sustainable agriculture and food waste valorization based on sustainability viewpoints. Regulatory standards and policy frameworks would be essential to strengthen to ensure the safety and effectiveness of phytochemical‐enriched foods. Public education and awareness programs would be necessary to bridge customer distrust and promote the uptake of phytochemical‐rich diets. In the future, phytochemicals can be fully exploited to foster environmental sustainability and world health by inspiring creativity and collaboration among scientific disciplines.

## Author Contributions

**Faiyaz Ahmed:** conceptualization (equal), writing – review and editing (equal). **Sammra Maqsood:** data curation (equal), formal analysis (equal), writing – original draft (equal). **Md Faruque Ahmad:** data curation (equal), formal analysis (equal), writing – review and editing (equal). **Ahmadullah Zahir:** conceptualization (equal), methodology (equal).

## Ethics Statement

The authors have nothing to report.

## Consent

All authors consent to publish this manuscript.

## Conflicts of Interest

The authors declare no conflicts of interest.

## Data Availability

The datasets generated, used, and/or analyzed during the current study are available from the corresponding author on reasonable request.
